# Farming Practice Influences Antimicrobial Resistance Burden of Non-Aureus Staphylococci in Pig Husbandries

**DOI:** 10.3390/microorganisms11010031

**Published:** 2022-12-22

**Authors:** Manonmani Soundararajan, Gabriella Marincola, Olivia Liong, Tessa Marciniak, Freya D. R. Wencker, Franka Hofmann, Hannah Schollenbruch, Iris Kobusch, Sabrina Linnemann, Silver A. Wolf, Mustafa Helal, Torsten Semmler, Birgit Walther, Christoph Schoen, Justin Nyasinga, Gunturu Revathi, Marc Boelhauve, Wilma Ziebuhr

**Affiliations:** 1 Institute of Molecular Infection Biology, University of Würzburg, 97080 Würzburg, Germany; 2 Department of Agriculture; South Westphalia University of Applied Sciences, 59494 Soest, Germany; 3 Genome Sequencing and Genomic Epidemiology, Robert Koch Institute, 13353 Berlin, Germany; 4 Advanced Light and Electron Microscopy (ZBS4), Robert Koch Institute, 13353 Berlin, Germany; 5 Institute of Hygiene and Microbiology, University of Würzburg, 97080 Würzburg, Germany; 6 Department of Pathology, Aga-Khan-University Hospital Nairobi, Nairobi, Kenya; 7 Department of Biomedical Sciences and Technology, The Technical University of Kenya, Nairobi, Kenya

**Keywords:** non-aureus staphylococci, NAS, alternative pig farming, antimicrobial resistance, one-health approach, intervention strategies, livestock-associated staphylococci, organic farming, pig farming methods

## Abstract

Non-aureus staphylococci (NAS) are ubiquitous bacteria in livestock-associated environments where they may act as reservoirs of antimicrobial resistance (AMR) genes for pathogens such as *Staphylococcus aureus*. Here, we tested whether housing conditions in pig farms could influence the overall AMR-NAS burden. Two hundred and forty porcine commensal and environmental NAS isolates from three different farm types (conventional, alternative, and organic) were tested for phenotypic antimicrobial susceptibility and subjected to whole genome sequencing. Genomic data were analysed regarding species identity and AMR gene carriage. Seventeen different NAS species were identified across all farm types. In contrast to conventional farms, no AMR genes were detectable towards methicillin, aminoglycosides, and phenicols in organic farms. Additionally, AMR genes to macrolides and tetracycline were rare among NAS in organic farms, while such genes were common in conventional husbandries. No differences in AMR detection existed between farm types regarding fosfomycin, lincosamides, fusidic acid, and heavy metal resistance gene presence. The combined data show that husbandry conditions influence the occurrence of resistant and multidrug-resistant bacteria in livestock, suggesting that changing husbandry practices may be an appropriate means of limiting the spread of AMR bacteria on farms.

## 1. Introduction

Antimicrobial resistance (AMR) in bacteria is a serious threat to public health with an estimated 4.95 million deaths worldwide in 2019 [1]. Highly concerning is the prediction that AMR could cause 10 million deaths annually by 2050, if left unaddressed [2]. Although the estimated numbers have been criticized as crude projections and apocalyptic, without considering the possible scientific inventions and advances of the future [3], AMR is nevertheless a rapidly growing threat to human health that requires immediate action. A systematic analysis of the global burden of AMR ranks methicillin-resistant *Staphylococcus aureus* (MRSA) as a common pathogen, causing 100,000 deaths just in 2019 [1]. Though MRSA is currently considered the clinically most important staphylococcal species, multidrug resistant (MDR) non-aureus staphylococci (NAS) play a significant role as opportunistic pathogens as well. Further, NAS are considered as an AMR gene source and reservoir for *S. aureus* and other pathogenic species. For example, *Mammaliicoccus sciuri* (formerly *Staphylococcus sciuri*) is likely to represent the evolutionary origin of methicillin resistance in staphylococci [4]. Hence, understanding the emergence of AMR in NAS is quintessential to prevent their further spread. A significant percentage of infections caused by AMR bacteria are attributed to inappropriate or overuse of antibiotics in agriculture through contamination of soil, water, livestock, and plant-derived foods, directly or indirectly [5,6]. The constant interplay between humans, livestock, and the environment in husbandry increases the likelihood of AMR transfer among different staphylococci and is thus contributing to the development of MDR organisms [7,8]. To fight the AMR issue in a coordinated manner, researchers around the world have agreed to pursue the One Health approach [9,10]. The One Health approach is defined as ‘the collaborative effort of multiple disciplines–working locally, nationally, and globally–to attain optimal health for people, animals and our environment’ [11].

The study presented here was performed within the framework of the “One Health PREVENT” consortium established by the National Research Network for Zoonotic Infectious Diseases, Germany to prevent the zoonotic spread of antibiotic-resistant pathogens [12]. Along with epidemiological studies to evaluate the spread of AMR in farm environments, the programme aimed at identifying suitable intervention strategies to curtail the spread of AMR. In this context, it was recently shown that straw bedding in combination with simple cleaning has a positive effect on reducing livestock-associated (LA)-MRSA colonisation in pigs [13,14]. Inspired by these findings, we analyse here the influence of housing conditions on the occurrence of antimicrobial resistance in commensal and environmental NAS in pig farms by phenotypic AMR testing and whole genome sequencing. For this purpose, we compared three farming methods (i.e., conventional, alternative, and organic) and focused on AMR in NAS that colonise pigs and sustain on barn surfaces. Depending on the antibiotic classes involved, we found fewer AMR-NAS in organic and alternative farms. Similarly, NAS carrying multiple acquired resistance determinants were rarer in organic and alternative farms than in conventional barns. The data suggest that husbandry conditions and farming practices may indeed have an impact on the occurrence of resistant bacteria in livestock.

## 2. Materials and Methods

### 2.1. Sampling of Environment and Animals

Forty-eight farms with fattening pigs, which include 24 conventional, 13 alternative, and 11 organic husbandries in Soest, Germany, were visited from March 2018 to September 2020. Conventional farming was defined by husbandry with a slatted or partially slatted floor in the bay, forced ventilation, and closed buildings. Alternative farming included husbandry on straw bedding, as well as outdoor climate or free ventilation in the stable, but with classification as conventional husbandry. Organic farming comprised straw bedding as well as outdoor climate or free ventilation in the stable. Further, the farms worked according to the guidelines for organic farming, as defined by relevant associations and organizations (i.e., Bioland e. V. (Mainz, Germany), Naturland e. V. (Gräfelfing, Germany). For commensal NAS collection, sampling per farm was performed from five randomly selected pigs per pen towards the end of the fattening period (10–12 weeks after stabling) by swabbing the pigs’ nostrils. For environmental NAS analyses, samples were taken from five to six different horizontal abiotic surfaces in the barn (with and without dust) as described previously [14]. Smear sets with liquid Amies transport medium (VWR, Langenfeld, Germany, #DELT300284) were used for nasal swabbing of the pigs. Swabs were inserted into one of the pig’s nostrils in circular movements, 2–3 cm deep without touching the outside of the snout. The environmental samples were taken with 10 × 5 cm sponges moistened with phosphate buffer (Medical Wire & Equipment, Corsham United Kingdom) over 30 cm and a total area of 300 cm^2^. All swabs were initially cultured at 37 °C for 18 ± 2 h in tryptic soy broth (TSB) (Merck, Darmstadt, Germany) with an addition of 6.5% sodium chloride (VWR, Langenfeld, Germany). After overnight culture, all samples were stored as 1:1 glycerol culture (87% glycerol; Bernd Kraft, Duisburg, Germany) at −80 °C.

### 2.2. Sample Isolation, Isolate Recovery, and Species Identification

For staphylococci isolation, each glycerol stock was streaked on mannitol salt agar (MSA) (Carl Roth, Karlsruhe, #CL81.1) and incubated overnight at 37 °C to obtain single colonies. Colonies were picked and subcultured on tryptic soy agar (TSA) (Carl Roth, Karlsruhe, Germany, #CP70.1). Single colonies were then patched in parallel on MSA and bile esculin agar (Sigma Aldrich, Darmstadt, Germany, #48300-500G-F) to detect and eliminate possible contamination with enterococci. After incubation on MSA, colonies which were able to ferment mannitol (indicated by colour change of MSA to yellow) were tested with Staphytect Plus (Oxoid, Wesel, Germany, #DR0850) to differentiate between *S. aureus* and mannitol-fermenting NAS. Finally, one NAS from each pig swab and three NAS from each surface swab were chosen at random to be analysed and stored at −80 °C as cryostocks. This led us to a total number of 240 NAS isolates. Species of the isolates were identified with automated mass spectrometry VITEK MS (bioMérieux Deutschland GmbH, Nürtingen, Germany) with Myla^®^ version 4.9.0–16 according to standard procedures provided by the manufacturer. According to manufacturer specifications, identifications with a confidence value of more than 60% were considered reliable.

### 2.3. Antimicrobial Susceptibility Testing

Minimum inhibitory concentrations (MIC) for cefoxitin, oxacillin, gentamicin, levofloxacin, erythromycin, clindamycin, linezolid, daptomycin, teicoplanin, vancomycin, tetracycline, tigecycline, fosfomycin, fusidic acid, rifampicin, and trimethoprim/sulfamethoxazole were determined using the VITEK2 Compact System (bioMérieux Deutschland GmbH, Nürtingen, Germany) according to standard procedures provided by the manufacturer (VITEK Card AST-P654, bioMérieux Deutschland GmbH, Nürtingen, Germany). MIC results were evaluated through the Advanced Expert System (AES)^TM^ of VITEK according to EUCAST guidelines (version 11.0) for NAS (https://www.eucast.org/clinical_breakpoints, accessed on 1 September 2022) [15]. Antibiotic susceptibilities for apramycin, spectinomycin, florfenicol, chloramphenicol, quinupristin-dalfopristin (quinupristin), and ciprofloxacin were performed by agar disc diffusion assays using commercially manufactured discs (Oxoid Deutschland GmbH, Wesel) with 15, 100, 30, 30, 15, and 5 µg of the respective antimicrobial agent according to EUCAST guidelines (version 11.0). *S. aureus* ATCC 29213 was used as a reference strain for disc diffusion assay. As neither clinical breakpoints nor epidemiological cut-off values applicable to staphylococci are available for apramycin, spectinomycin, and florfenicol, inhibition zone distributions were determined (Figure S1).

### 2.4. DNA Extraction and Whole Genome Sequencing (WGS)

NAS isolates were cultured on Columbia sheep blood agar (Mast Diagnostica, Reinfeld, #201190) and DNA was extracted using the NucleoSpin Tissue Kit (Macherey-Nagel, Dueren, #740952), according to the manufacturer’s protocol, adding 15 μL lysostaphin (2 mg/mL) to the lysis buffer. A total of 240 samples spanning 17 different species of staphylococci were sequenced and computationally analysed. Isolates were Illumina sequenced on a NextSeq 2000 platform to generate paired-end reads of 150bp length. Matching metadata including species, farming type, and sampling location were available for each isolate. Raw sequencing reads were uploaded to the Sequence Read Archive (SRA) of NCBI and are available under BioProject ID (PRJNA903486).

### 2.5. Phylogenetic Analyses

Raw sequencing reads were first adapter- and quality-trimmed using an in-house pipeline, and further assembled into contigs through the use of the SPAdes genome assembler (v3.13.3) [16] using the “--careful” option. Core genome Multi Locus Sequence Typing (cgMLST) was performed on these contigs through the utilisation of the chewBBACA tool (v2.8.5) [17]. Gene prediction was performed within chewBBACA and the resulting cgMLST scheme, NASisting of 869 core genes shared by 90% of the isolates, was concatenated in order to compute a multiple sequence alignment through MUSCLE (v3.8.1551) [18]. Phylogenetic relationships were then assessed through further analysis with RAxML-NG (v0.9.0) [19] using the GTR+G model, 10 randomized parsimony starting trees, and 20 bootstrap replicates. The resulting Newick tree was visualised with the corresponding metadata in iTOL [20]. This workflow was written in Python (v3.7.12) and is publicly available under GLPv3 license on GitLab for reproducibility (https://gitlab.com/mustah98/coregenomephylo, accessed on 1 July 2022).

### 2.6. Genotypic Antibiotic Resistance Analyses

Assembled strains were further computationally screened for genes conferring resistance to antibiotics using AMRFinderPlus (v3.10.18) [21]. Samples were hereby compared to the NCBI Bacterial Antimicrobial Resistance Reference Gene Database (PRJNA313047) and gene hits with a query coverage and identity >90 % each were summarised and visualised. AMR genes and their corresponding classes were visualised as heatmaps and bar charts using R (4.1.0). Genomic annotation was performed for all strains using Prokka (v1.1.14) [22] with the genus set as ‘*Staphylococcus*’. The utilised antibiotic resistance and annotation analysis workflow, named ARpip, was written in Python (v3.7.12) and is publicly available under the GLPv3 license on GitLab (https://gitlab.com/mustah98/ARpip, accessed on 1 July 2022).

### 2.7. Statistics

Pearson’s chi-square test was used to compare the relative abundances of the detected staphylococcal species in samples from conventional, alternative, and organic agriculture, respectively. For individual species and resistance analyses, the two-sample test for equality of proportions with continuity correction was used as implemented in the statistics software R vers. 4.2.1 [23]. Alternative and organic farms were compared each with conventional farms as the baseline. The proportion of resistant isolates was compared for 22 antibiotics individually at the phenotypic level using the two-sample test for equality of proportions with continuity correction. To assess the statistical significance of the pairwise comparisons between alternative and organic farming with conventional farming, the Bonferroni multiple test correction method was used for *n* = 2 × 22 = 44 comparisons. Accordingly, adjusted *p*-values smaller than 0.0011 were considered statistically significant for each antibiotic and comparison. Likewise, for the 11 antibiotic resistances compared at the genotypic level, *p*-values smaller than 0.0022 were considered statistically significant; for the 17 NAS species tested, *p* < 0.03 was set as the cut-off.

## 3. Results

### 3.1. Species Detection and Distribution

**Figure 1:**
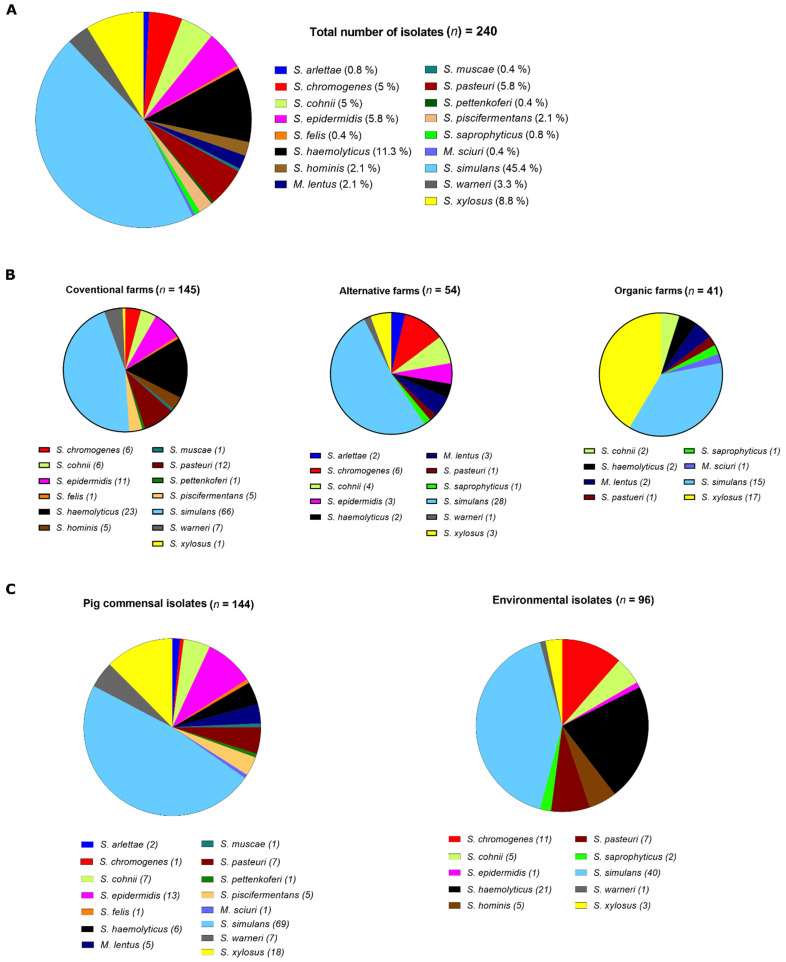
Distribution of NAS isolates in different farm types (*n* = 240) (**A**) NAS species detected in the pigs and the farms. (**B**) Classification of NAS isolates according to their farming type. (**C**) Classification of NAS isolates according to their sampling site.

NAS isolates were recovered from nasal and surface swabs through culturing in a series of different selection media as described in the method Section 2.2. Initial species identification was performed by automated mass spectrometry (MS) by employing the VITEK-MS System which detected 17 different species in the sample, with species distribution being equally diverse between farm types (Figure 1A, Table S1). *S. simulans* was widespread in conventional (45.5%), alternative (51.8%), and organic (36.5%) husbandries, while *S. xylosus* (41.4%) was also common in organic farms (Figure 1B, Table S1). The isolates were also analysed based on their sampling location, either as animal commensals from pigs (*n* = 144) or as environmental isolates from abiotic barn surfaces (*n* = 96) (Figure 1C). Again, *S. simulans* was common in both groups. Interestingly, some species such as *S. haemolyticus* and *S. chromogenes* were common in environmental samples (21.9%; 11.5%), while their occurrence was significantly rarer in animal samples (0.04%; 0.006%) (Figure 1C, Table S1). Conversely, *S. xylosus* and *S. epidermidis*, the second (12.5%) and third (9%) most common species of animal origin, were rarely detected in surface samples (3.1% and 1%, respectively) (Figure 1C, Table S1).

### 3.2. Phenotypic Resistance Profiles of NAS Isolates

**Figure 2:**
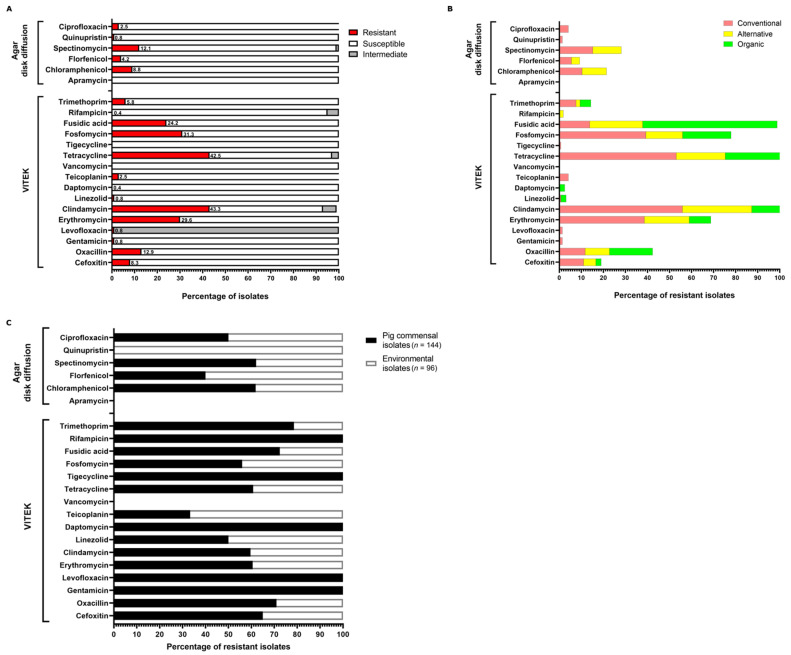
Phenotypic antibiotic resistance determination by VITEK and agar disk diffusion. (**A**) Resistance profile of all NAS isolates (*n* = 240). The percentage of resistant (red), susceptible (white), and intermediate (grey) isolates are represented by the x-axis (**B**) The assignment of the resistant isolates to the individual farm types is shown in a bar chart: conventional (red), alternative (yellow), and organic (green). (**C**) Categorisation of resistant isolates according to their site of sampling, as either pig commensal isolates (black bar) or environmental isolates (white bar).

Phenotypic characterisation of antibiotic resistance profiles of the isolates was carried out using the VITEK2 Compact System and agar disk diffusion assays. The antibiotic susceptibility data for each isolate are provided in Table S2. Resistance of the NAS isolates against antibiotics commonly used in human and veterinary medicine was tested, and the results are presented in Figure 2. A number of the total isolates exhibited resistance against clindamycin (106/240; 43.3%), erythromycin (71/240; 29.6%), tetracycline (102/240; 42.5%), fosfomycin (75/240; 31.3%), fusidic acid (58/240; 24.2%), oxacillin (13/240; 5.4%), and aminoglycosides (31/240; 12.9%), whereas resistance against last-resort antibiotics such as vancomycin (0/240), daptomycin (1/240; 0.4%), and linezolid (2/240; 0.8%) was rare in the sample and occurred in various species both in conventional and organic farms (Figure 2A,B). Phenotypic resistance patterns classified by farm type are shown in Figure 2B and Table S3. For most antibiotics tested, the majority of resistant isolates were from conventional farms, while alternative and organic farm isolates were associated with fewer AMR (Figure 2B). An exception to this pattern was resistance to fusidic acid. Here, more isolates (25/41; 60.9%) from organic farms were fusidic acid resistant, compared to 20/145 (13.7%) of the conventional farm isolates (*p* = 1.721 × 10^−9^) (Figure 2B and Table S3). The most common AMR phenotypes in conventional farms were detected against tetracycline (77/145; 53.1%), clindamycin (81/145; 55.8%), erythromycin (56/145; 38.6%), and fosfomycin (57/145; 39.3%). Statistically highly significant differences between conventional and organic farm isolates were detected for resistance to erythromycin (56/145 vs. 4/41; *p* = 0.000961) and clindamycin (81/145 vs. 6/41; *p* = 6.985 × 10^−6^), as well as for tetracycline resistance in the alternative farm isolates (*p* = 0.0001873) (Figure 2B and Table S3). Phenotypic aminoglycoside resistance occurred in 24/145 (16.5%) of the conventional farm isolates and was mainly driven by resistance to spectinomycin which was detected in 22/24 (91.7%) of the resistant isolates. In contrast, aminoglycoside resistance was absent in organic farm isolates (0/41) and rare (7/54; 12.9%) in alternative farms, with the differences, however, not reaching statistical significance (Figure 2B and Table S3). Similarly, oxacillin/cefoxitin resistance was recorded in 15/145 (10.3%) isolates from conventional farms, while the phenotype was present in only 1/41 (2.4%) and 2/54 (3.7%) of the organic and alternative farm isolates, respectively (Figure 2B and Table S3). For most of the 22 compounds tested, we found at least one resistant isolate among the conventional farm isolates (i.e., 18/22). In contrast, alternative and organic farms’ isolates were phenotypically resistant to only 12 and 10 of the 22 compounds tested, respectively (Figure 2B). Analysis of the isolates according to their site of sampling, either as samples from pig nostrils or as surface samples in the barn, indicated that resistance is mainly driven by the commensal animal-derived isolates and to a lesser extent by surface isolates from the environment (Figure 2C). Exceptions are resistances against teicoplanin, florfenicol, and quinupristin. Here, resistant isolates originated predominantly from the environment. Strikingly, resistances against gentamicin, levofloxacin, daptomycin, tigecycline, and rifampicin were exclusively exhibited by pig commensals, but not by dust-derived isolates (Figure 2C).

### 3.3. Genome Sequencing and Phylogenetic Analyses

**Figure 3:**
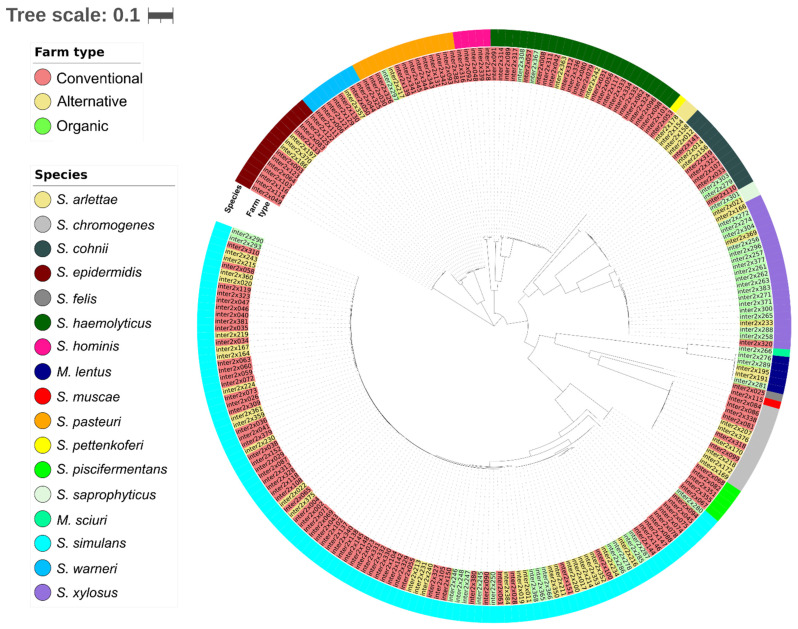
Phylogenetic and population analyses using iTOL: Phylogenetic tree displaying the species distribution among the farm isolates. In the inner ring, the isolates are grouped according to their closeness and are colour-coded representing their farm type; conventional (red), alternative (yellow), and organic (green). The outer ring represents the classification of species for the isolates.

All isolates were subjected to Illumina whole genome sequencing and analysed as described in method Section 2.5. The evolutionary relatedness of the isolates was assessed through phylogenetic reconstruction, and the resulting tree was visualised within iTOL version 6.6 [20] (Figure 3). The results of this analysis are depicted as a phylogenetic tree shown in Figure 3. The computational analysis grouped the isolates species-wise with zero outliers and confirmed the species identification data obtained by VITEK-MS.

### 3.4. Genotypic Resistance Profiles of Isolates

**Figure 4:**
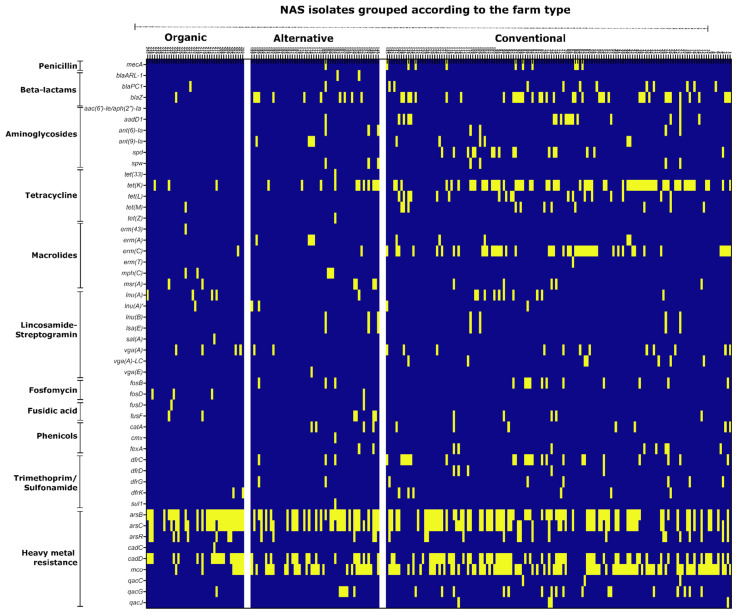
Genotypic resistance profile of the isolates. Heat map representation of the presence (yellow) or absence (blue) of different antimicrobial genes (AMR) screened. The x-axis depicts the arrangement of isolates according to the farm type; on the y-axis, the screened antimicrobial resistance genes are arranged according to their antibiotic class.

The genome sequences obtained were screened for the presence of known antimicrobial resistance genes using AMRFinderPlus (v3.10.18) as described in method Section 2.6. The AMR genes were grouped according to the class of antibiotic they confer resistance to. Likewise, isolates were sorted according to their origins from the different farm types. The results from these analyses are summarised in the heatmap shown in Figure 4 and details are given in Table S3. Depending on the antibiotic class analysed, we found remarkable differences regarding AMR carriage between farm types. With respect to **beta-lactams**, the methicillin-resistance mediating *mecA* gene was absent in the 41 organic farm isolates and was rare in isolates from alternative farms (1/54; 1.8%), while *mecA* was present in 10/145 (6.9%) of the conventional farm isolates, although differences did not reach statistical significance (Table S3). Further, the beta-lactamase genes *blaZ and blaPC1* occurred frequently among isolates from conventional farms (49/145; 34%). Of the alternative farm isolates, 13/54 (24%) carried either *blaZ, blaPC1* or *blaARL-1*, while *blaZ* and *blaPC1* were detected in only two organic farm isolates (2/41; 4.9%) (Figure 4; Table S3). **Aminoglycoside** resistance genes, present in the samples, comprised *ant(9)-Ia*, *ant(6)-Ia, spw* and *spd* as well as *aadD1* and *aac(6′)-Ie/aph(2′’)-Ia* (Table S3). These genes were exclusively detected in conventional and alternative farm isolates with 35/145 (24.1%) of the conventional and 7/54 (12.9%) of the alternative farm isolates carrying at least one of these aminoglycoside resistance genes. In contrast, aminoglycoside resistance genes were absent in the 41 isolates from organic farms, demonstrating significantly less aminoglycoside AMR gene presence in comparison to the conventional farm isolates (*p* = 0.001094). (Figure 4; Table S3). Other resistance genes which were absent or rare in the genomes of organic farm isolates comprised AMR genes against **phenicols** (0/41) and **trimethoprim** (i.e., *dfrK*, 2/41; 4.9%), while such genes were more common in conventional and alternative farm isolates, with differences, however, not reaching statistical significance (Figure 4; Table S3). Moreover, in conventional farms, a wide range of AMR genes mediating **tetracycline** resistance were detected, which comprised *tet(K), tet(L), tet(M)* and *tet(Z)*. From the 145 conventional farm isolates, 80 (55.2%) carried at least one or more of these genes, and in 11/54 (20.3%) of the alternative farm isolates, tetracycline resistance genes were detected, which were mainly *tet(K).* In contrast, only a few isolates from organic farms (4/41; 9.7%) harboured tetracycline AMR genes (i.e., *tet(K), tet(M)*). Related to the conventional farm isolates, these differences were statistically significant for both the alternative (*p* = 2.421 × 10^−5^) and organic farm isolates (*p* = 6.301 × 10^−7^) (Figure 4; Table S3). Regarding **macrolide resistance**, we found a broad range of AMR genes with *erm(C)* being the most abundant one (Figure 4; Table S3). Among the conventional and alternative farm isolates, 52/145 (35.9%) and 12/54 (22.2%) carried at least one macrolide AMR gene, respectively, while 5/41 (12.2%) of the organic farm isolates harboured a macrolide resistance gene. The difference between conventional and organic farms was striking but was not highly statistically significant (*p* = 0.006719) (Figure 4; Table S3). **Lincosamide-Streptogramin** AMR genes were equally detected across all three farm types, with 33/145 (22.7%) of the conventional, 9/54 (16.7%) of the alternative, as well as 9/41 (21.9%) of the organic farm isolates carrying at least one lincosamide-streptogramin AMR gene (Figure 4; Table S3). Similarly, no difference between farm types was detectable regarding AMR gene carriage against fosfomycin and fusidic acid, as the associated AMR genes were found to be evenly distributed among the isolates (Figure 4). In addition to clinically relevant antimicrobials, we also analysed the genomes with respect to resistance to heavy metals and disinfectants. Genes that code for resistance against heavy metals, such as arsenic (*arsB*, *arsC*, *arsR*), cadmium (*cadC*, *cadD*), and copper (*mco*) were widespread among the isolates, irrespective of their origin from different farming types (Figure 4 and Table S2). The presence of genes encoding resistance to quaternary ammonium compounds (*qacC*, *qacG*, *qacJ*) was relatively low in the sample (29/240; 12%), especially among the organic farm isolates (1/41; 2.4%).

### 3.5. Multiple Resistance Determinant (MRD) Analysis

Isolates harbouring multiple resistance determinants (MRD) were defined by displaying resistance to more than two distinct antimicrobial classes. Both phenotypic and genomic AMR analyses of isolates were performed to identify MRD isolates, excluding species-dependent intrinsic resistance.

**Figure 5:**
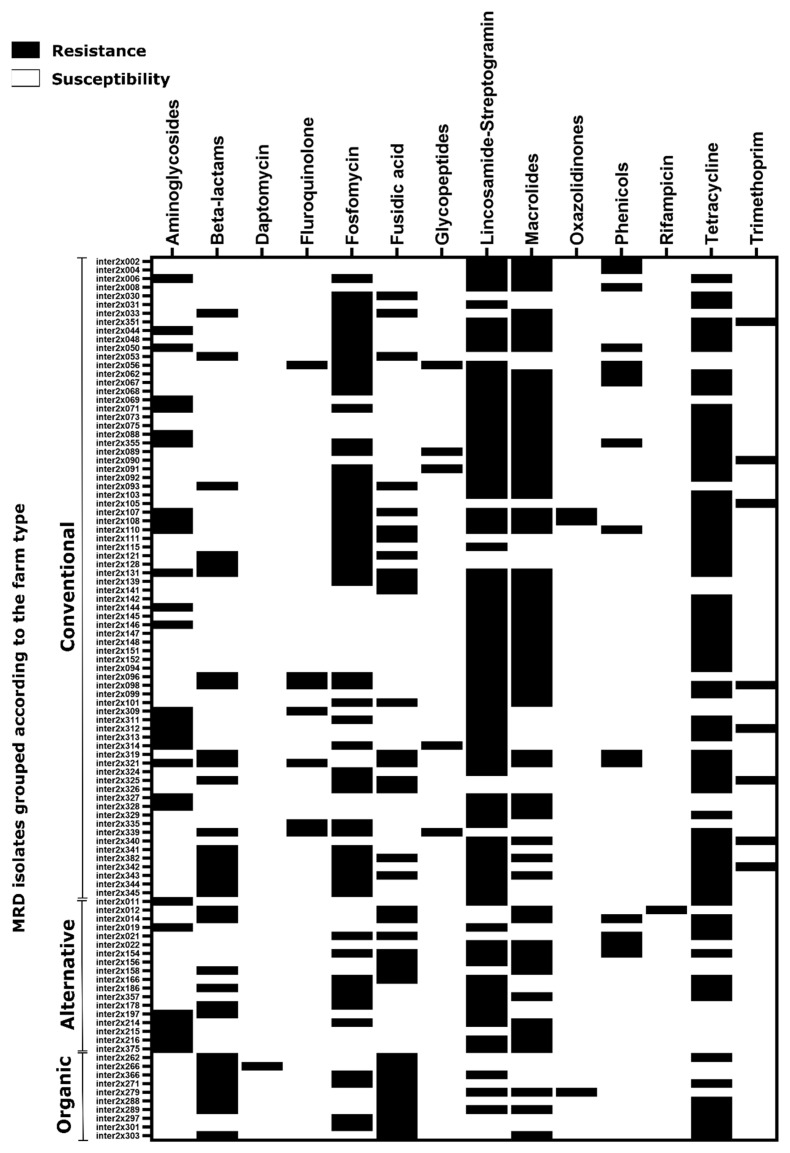
Isolates harbouring multiple resistance determinants (MRD). Heat map representation of the MRD isolates and their resistance against different antibiotic classes tested. On the y-axis, the MRD isolates are classified according to farm type.

The phenotypic resistance data, obtained by VITEK and disc diffusion assays, revealed that 42% (*n* = 102) of the total isolates represent MRD isolates. Furthermore, the resistance data of the MRD isolates were plotted according to the farm type, and the results are displayed as a heatmap in Figure 5. The majority of the MRD isolates belonged to conventional farms (*n* = 74), followed by alternative (*n* = 18), and organic farms (*n* = 10), and the differences between conventional and both alternative (*p* = 0.03872) and organic (*p* = 0.004383) farm isolates were statistically significant (Table S3). Farm-wise analysis revealed that 51% of the conventional farm isolates were MRD, whereas this percentage was lower in the case of alternative (33.3%) and organic farms (24.3%) (Table 1). Similarly, when analysing the data set according to genomic AMR gene detection, we detected MRD in 51% (74/145) of the conventional, in 27.7% (15/54) of the alternative, and in 9.8% (4/41) of the organic farm isolates, again detecting significant differences between conventional and both alternative (*p* = 0.005542) and organic (*p* = 5.363 × 10^−6^) farm isolates (Table 2). The results of this analysis are summarised in Table S3 and illustrated as a heatmap in the supplementary (Figure S2). The combined phenotypic and genotypic analyses demonstrate that MRD presence in conventional farms was at least two times higher than in organic farms.

## 4. Discussion

NAS are not only normal ubiquitous bacteria in hospital and livestock-associated environments, but also represent sources of transferable antimicrobial resistance genes and virulence factors [24,25,26]. This study analysed the influence of different farming methods on the AMR burden in commensal and environmental NAS in pig farms. Precisely, we tested whether differences exist between conventional, alternative, and organic pig husbandries, with respect to AMR-NAS presence. Conventional holding conditions comprised keeping the pigs on the slatted floor and under closed ventilation, whereas alternative and organic farms employed straw bedding in combination with an outdoor climate and/or free ventilation. The organic farms additionally adhered to the rules and regulations of their associations on feeding and the restrictive use of antibiotics [27,28]. In order to get an insight into the resistance situation in non-infection-associated staphylococci, we randomly recovered commensal NAS from nasal swabs of pigs, as well as NAS from the environment in the barns (i.e., abiotic horizontal surfaces). Species characterisation of the 240 NAS isolates obtained was performed by VITEK-MS and was further confirmed by whole genome sequencing. *S. simulans* was the dominant species in the study. The species is an opportunistic animal pathogen and causes bovine mastitis in cattle and endocarditis in birds [29,30,31]. Recently, *S. simulans* has also been implicated in human infections related to diabetes and prosthetic joints, particularly in individuals with regular contact with farm animals [32,33,34,35]. *S. simulans* was previously detected in livestock and livestock-associated environments worldwide [36,37,38,39,40], and also in our study *S. simulans* was widespread in all three housing systems (Figure 1). Although not reaching statistical significance, the common occurrence of *S. xylosus,* particularly among the organic farm isolates, was interesting as well (Figure 1, Table S1). As a NAS species, *S. xylosus* is gaining interest due to its increasing clinical appearance and frequent association with animal/human infection and serious multidrug resistance [41,42,43,44]. *S. xylosus* is a common commensal, originally isolated from the skin of animals and humans [45,46], and the species is reported to be frequently isolated from livestock and farm environments [47,48,49]. A link between *S. xylosus* and organic farming has not yet been established, although some reports seem to point in this direction. Thus, Roberts et al. 2018 observed *S. xylosus* to be more frequently present in organic dairy farms in Washington State, when compared to conventional farms included in the study. However, this finding was not discussed elaborately in the article [50]. Likewise, a study in Mexico reported *S. xylosus* as the major species isolated from backyard farm animals [47]. Currently, we have no biological rationale for the *S. xylosus* presence in organic farms in our study. One explanation could be the combination of free roaming and organic feeding of pigs with straw bedding, with the latter providing abundant xylose which is a major component of plant materials and wood [51,52]. As the name suggests, *S. xylosus* is capable of efficiently utilising xylose as a carbon source [53,54,55] which could give the species a metabolic advantage over other NAS.

Over the years, it was well established that pigs and pig farm environments are reservoirs of AMR NAS [40,56,57]. These studies were mainly performed in conventional farms, revealing high AMR rates against many commonly used antibiotics. Here, we compared AMR patterns in NAS from different housing conditions. In general, we detected less AMR in NAS from alternative and organic farms than in conventional farms, but this was highly dependent on the antibiotic class studied. Additionally, we observed, for some antibiotics, discrepancies between phenotypic resistance and corresponding AMR gene detection in the NAS genomes. Regarding **beta-lactam resistance**, *mecA* carriage was exclusively detected in conventional and alternative farm isolates but was completely absent in NAS from organic farms (Figure 4; Table S3). As *mecA* gene carriage is considered (both in *S. aureus* and NAS) as a marker for isolates of concern, this is an interesting finding. All *mecA*-positive isolates displayed simultaneous resistance to oxacillin and cefoxitin which is the canonical resistance phenotype mediated by the *mecA*-encoded alternative penicillin binding protein PBP2a, conferring low affinity to all beta-lactams. Interestingly, however, we also found a number of NAS isolates in which phenotypic beta-lactam resistance and AMR gene detection did not match. We neither detected *mec* genes nor any of the currently known beta-lactamase genes in four isolates displaying oxacillin/cefoxitin resistance, in two isolates displaying sole cefoxitin resistance, as well as in thirteen isolates that were exclusively oxacillin resistant (Table S3). Generally, aberrant beta-lactam resistance in staphylococci is not unusual, and particularly methicillin resistant staphylococci which lack *mec* genes (MRLM) have been described [58]. The non-canonical resistance pattern in MRLM has been associated with several mechanisms such as deregulation and overexpression of *blaZ* genes [59], low beta-lactam affinity of native PBPs due to mutations [60], and forced peptidoglycan cross-linking upon PBP4 overexpression, caused by mutation of the *pbp4* promoter [61]. Finally, mutations in the *gdpP* gene, mediating second messenger cyclic di-AMP synthesis, were recently also associated with MRLM in *S. aureus* [62]. To determine which of these mechanisms is responsible for the MRLM phenotype in the NAS isolates, will require further investigations. Other AMR genes that occurred exclusively in isolates from conventional and alternative farms, but were absent in organic farms, included AMR against aminoglycosides and phenicols. Phenotypic **aminoglycoside** resistance was mainly determined by spectinomycin resistance, and we found a broad spectrum of spectinomycin AMR genes predominantly in the isolates from conventional husbandry (Figure 4 and Table S3). Spectinomycin is approved for application in food-producing animals and is commonly used for the treatment and metaphylaxis of dysentery in pigs, where it is administered orally (in combination with lincomycin) through the drinking water [63]. Although we do not have specific information on whether participating farms used spectinomycin during the study period, it is reasonable to speculate that the aforementioned widespread practice in conventional pig farming may have influenced the occurrence of spectinomycin AMR genes [63]. A similar circumstance may apply to **phenicols**, of which florfenicol is approved for use in food-producing animals and is commonly used to treat respiratory tract infections in pigs [64]. Phenicol AMR genes were exclusively detected among conventional and alternative farm isolates and comprised mainly *fexA*, encoding a florfenicol/chloramphenicol efflux transporter, and *catA* which specifically inactivates chloramphenicol, but not florfenicol [64]. The *catA* gene occurred either alone or in combination with *fexA* which is surprising as chloramphenicol is not used in food-producing animals, suggesting a low selection pressure for *catA* in the NAS genomes. However, since *cat* genes are usually encoded on plasmids, we consider co-selection with other plasmid-encoded traits a likely scenario for maintaining the gene in the population. Consistent with previous studies on AMR in NAS isolates from livestock [40,56], the isolates in our study had high rates of resistance to **tetracyclines** and **macrolides**, with the majority of resistant isolates again coming from conventional and alternative farms. The use of tetracycline and macrolide antibiotics in swine husbandry and veterinary medicine is well documented [65,66]. Evidence of AMR to these antibiotics is, therefore, not surprising and may be associated with high selection pressure. The most common **tetracycline** AMR genes in the sample were *tet(K)* and *tet(L)*, encoding tetracycline transporters, as well as *tet(M)* which codes for a ribosomal protection protein, with a few isolates even harbouring more than one of these genes. *Tet* genes may be linked to macrolide-lincosamide-streptogramin (MLS) resistance genes on transposable elements, reflecting once again selection pressure from the frequent use of tetracyclines and macrolides [67]. In fact, **macrolide** AMR gene detection was also common among conventional farm isolates. Here, the most abundant macrolide AMR gene was the macrolide-lincosamide-streptogramine B (MLS_B_) resistance-mediating *erm©* which occurred in 45/145 (31%) of the isolates from conventional farms, but only in one isolate each from alternative and organic farms, respectively. We also noticed AMR genes conferring resistance to **lincosamides** (i.e., *lnu(A)*, *lnu(A)’*, *lnu(B)*) and lincosamides-streptogramin A (LS_A_) (i.e., *vga(A)*, *vga(A)-LC*, *vga(E)*, *sal(A)*) in the sample. While clindamycin is not approved for application in food-producing animals, the lincosamide antibiotic lincomycin is commonly used in combination with spectinomycin for treatment and metaphylaxis of pigs (see above) and was previously employed as a growth promoter in livestock [68]. At first glance, we found no significant difference in the detection of lincosamide AMR genes between the different farm types (Figure 4; Table S3). However, closer examination of the data set revealed that detection of lincosamide AMR genes in conventional and alternative farm isolates was often associated with the simultaneous presence of spectinomycin AMR genes, whereas lincosamide AMR genes occurred singly in isolates from organic farms (Table S2). It is reasonable to speculate that the application practice of lincosamides/spectinomycin in conventional animal husbandry may have contributed to co-selection with spectinomycin resistance traits. Regardless of their origin from different farm types, AMR to **fosfomycin** and **fusidic acid** was also common among NAS isolates. Fosfomycin is a peptidoglycan synthesis-targeting antibiotic which is not approved in the European Union in food-producing animals and is restrictedly used in veterinary medicine [63]. Of the 75/240 phenotypically fosfomycin-resistant NAS, 18 carried transmissible fosfomycin resistance genes (i.e., *fosB*, *fosD*). Apart from species-associated intrinsic resistance in a few isolates (e.g., in two *S. saprophyticus*) the genetic mechanisms of fosfomycin resistance in the other isolates (e.g., by mutations in chromosomal genes) remain to be determined [69,70]. We currently have no plausible explanation for the relatively high fosfomycin resistance rate in the samples, but the data are consistent with a previous study, reporting frequent fosfomycin resistance in NAS as well [40]. Deviating from the usual pattern with more resistances in the conventional farms, we observed more phenotypic resistance to **fusidic acid** in the organic farm isolates (Figure 4; Table S3). Fusidic acid binds and inhibits elongation factor G (encoded by *fusA*) and is mainly used topically to treat staphylococcal skin infections in humans and companion animals [71,72]. Genetic determinants attributed to fusidic acid resistance were reported among clinical and farm isolates of staphylococci [73,74,75,76]. In our sample, we were able to reveal the genetic mechanism for the fusidic acid resistance phenotype in 14/58 resistant isolates which was exclusively due to factors intrinsic to distinct NAS species. Thus, two *fusD*-positive isolates were *S. saprophyticus* which carry *fusD* intrinsically, and another 12 isolates represented *S. cohnii*, known to harbour intrinsic *fusF* [6,77]. We did not detect any of the known transferrable *fus* resistance genes (i.e., *fusB*, *fusC*), which may reflect the low selective pressure by this antibiotic in pig farming. Elucidation of the molecular background of fusidic acid resistance in the other resistant strains (e.g., by *fusA* mutations) will nevertheless be interesting and justify more detailed investigations. Of note, three isolates in the sample displayed phenotypic resistance against the last-resort antibiotics **daptomycin** and **linezolid**, respectively (Figure 2). Emerging resistance to last-choice antibiotics is considered a serious threat to public health, and is therefore monitored with concern [1,78]. While daptomycin resistance is primarily associated with adaptive chromosomal mutations [79], a number of acquired and mobile resistance genes such as *cfr*, *optrA*, and *poxtA* have been identified that mediate linezolid resistance, and in the last decade resistance against linezolid in NAS has emerged with a reported global rate of <2% [80,81,82,83]. Although linezolid is not approved for veterinary use, the resistance genes seem to sustain and circulate in animal husbandry, as exemplified by the detection of *cfr* in LA-MRSA [80,81,82]. This might be associated with cross resistance to other antimicrobials (e.g., phenicols, lincosamides). In our study, however, none of the isolates carried a known linezolid resistance gene, indicating that more detailed molecular work is required to elucidate the underlying mechanisms in the linezolid resistant NAS detected in this study. We also noticed frequent detection of heavy metal resistance (HMR) genes among the isolates, irrespective of the farm type. The data are in good agreement with a previous study on LA-MRSA, showing that 75.3% of the isolates carried at least one HMR gene [84]. Co- and cross resistance between metals and antimicrobials is well documented and is probably associated with the use of heavy metals in farming environments as fertilisers, animal feeds, and disinfection agents, but may also reflect the natural exposure of bacteria in soil environments to metals [85,86,87]. Finally, the frequent detection of isolates carrying multiple (acquired) resistance determinants (MRD), especially on conventional farms, once again underscores the potential of NAS as AMR gene reservoirs. The lower MRD-NAS prevalence in organic farms suggests that it is possible to reduce the resistance potential of these strains.

## 5. Conclusions

Meanwhile, the correlation between exposure to antibiotics and the emergence and selection of AMR bacteria in animal husbandry is beyond question [6]. Therefore, significant efforts are being made to reduce the use of antibiotics in food production and improve antibiotic waste management to avoid environmental pollution [88,89]. However, to really tackle the current resistance problem in agriculture, further effective measures are needed, such as antibiotic surveillance and stewardship, as well as adaptation of husbandry conditions [14]. Regarding the latter, only a limited number of studies addressed the influence of husbandry practices on the spread of AMR in livestock isolates so far [13,39,90,91]. With this study, we aimed at filling this knowledge gap. Indeed, we found that NAS from conventional farms frequently harboured AMR genes against antibiotics that are also commonly used in human medicine, while the occurrence of critically resistant NAS isolates was much lower or even absent in organic farms. Isolates from the alternative farms often occupied an intermediate position between conventional and organic farms in terms of AMR frequency, which raises the question of differences particularly between organic and alternative farms. Both farm types use straw bedding and free ventilation in the barns, but organic farms additionally adhere to the strict guidelines of their associations, which require the feeding of organically produced feed and regulate the extremely restrictive use of antibiotics. Based on the data obtained, we consider straw bedding as the critical factor that significantly lowers the AMR rates, both in the alternative and organic farms, compared to slatted floor keeping (and closed ventilation) in the conventional husbandries. Although the specific causal relationships are not yet clear, it is reasonable to speculate that the microbiota on the straw increase diversity of the bacterial communities in the barns. As antibiotic resistance acquisition usually comes at a fitness cost, it is likely that the straw microbiota competes with AMR bacteria and displace them successfully from the ecological niche. Avoidance of antibiotics and organic feeding may then further reduce AMR prevalence in the organic farms. Clearly, more experimental work is required to substantiate this hypothesis, but the data obtained so far suggest that this might be a likely scenario. If it holds true, straw bedding will represent a very simple (and traditional) measure, not only to effectively lower the AMR burden in livestock, but to contribute to animal well-being as well. Altogether, the study shows that changes in farming practice have a huge potential to curtail the emergence and spread of AMR bacteria in agriculture.

## Figures and Tables

**Table 1:** Origin of phenotypic MRD isolates (*n* = 102) displaying resistance against more than two different antimicrobial classes.

Farm Type	Total Number of Isolates in the Farm-Type	Number of MRD Isolates	Percentage of Isolates that Were MRD
Conventional	145	74	51
Alternative	54	18	33.3
Organic	41	10	24.4

**Table 2:** Origin of genotypic MRD isolates (*n* = 93) carrying more than two AMR genes against different antimicrobial classes.

Farm type	Total Number of Isolates in the Farm-Type	Number of MRD Isolates	Percentage of Isolates that Were MRD
Conventional	145	74	51
Alternative	54	15	27.8
Organic	41	4	9.7

## Data Availability

The genome sequencing data were uploaded to the Sequence Read Archive (SRA) of NCBI (Home-SRA-NCBI (nih.gov)) and are available under BioProject ID (PRJNA903486).

## Supplementary Materials

The following supporting information can be downloaded at: https://www.mdpi.com/article/10.3390/microorganisms11010031/s1. Figure S1: Inhibition zone distributions for apramycin (APR), spectinomycin (SPC), florfenicol (FFC) to determine the resistant isolates. Table S1: Species distribution in the population. Table S2: Metadata as an excel file comprising the results of phenotypic antibiotic susceptibility test and genotypic screening of antimicrobial resistance genes for all the isolates. Table S3: (i) Number of isolates displaying phenotypic and genotypic resistance for the antibiotic classes tested (ii) Statistical analysis data of phenotypic and genotypic resistance distribution in the different farm types *. Figure S2: Antibiogram of the isolates harbouring multiple resistance determinants (MRD) in the population.
