# Supplementary material for: Farming Practice Influences Antimicrobial Resistance Burden of Non-Aureus Staphylococci in Pig Husbandries
Source: Microorganisms. 2022 Dec 22;11(1):31. doi: 10.3390/microorganisms11010031 (PMC9865537; doi:10.3390/microorganisms11010031)
Supplement: Supplementary file 1 [file microorganisms-11-00031-s001.zip › microorganisms-2101959-supplementary-main.pdf]

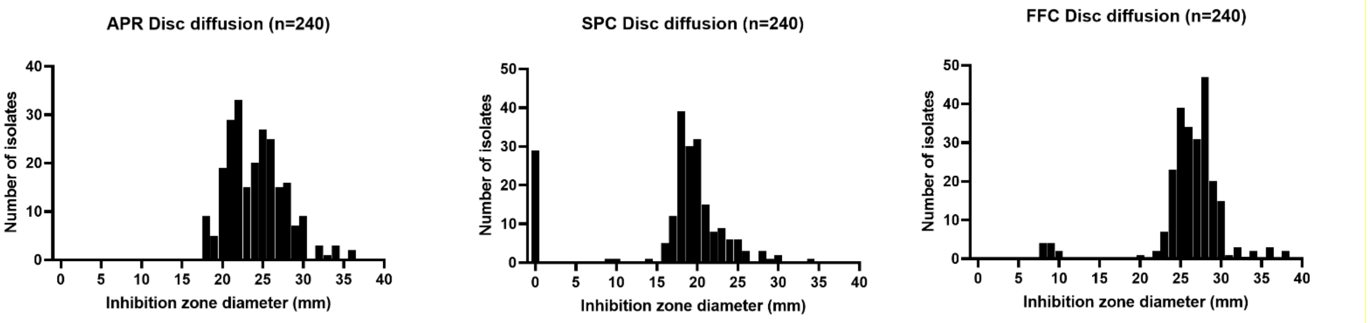

Figure S1. Inhibition zone distributions. The inhibition zone diameters (mm) of the NAS isolates are depicted in the x-axis of the graph. Depending on the diameter of the inhibition zone, the isolates were classified as resistant (R), susceptible (S) or intermediate (I) for each antibiotic tested: SPC (R: 0 mm; I: 9 to 10 mm), FFC (R: 8 to 10 mm).

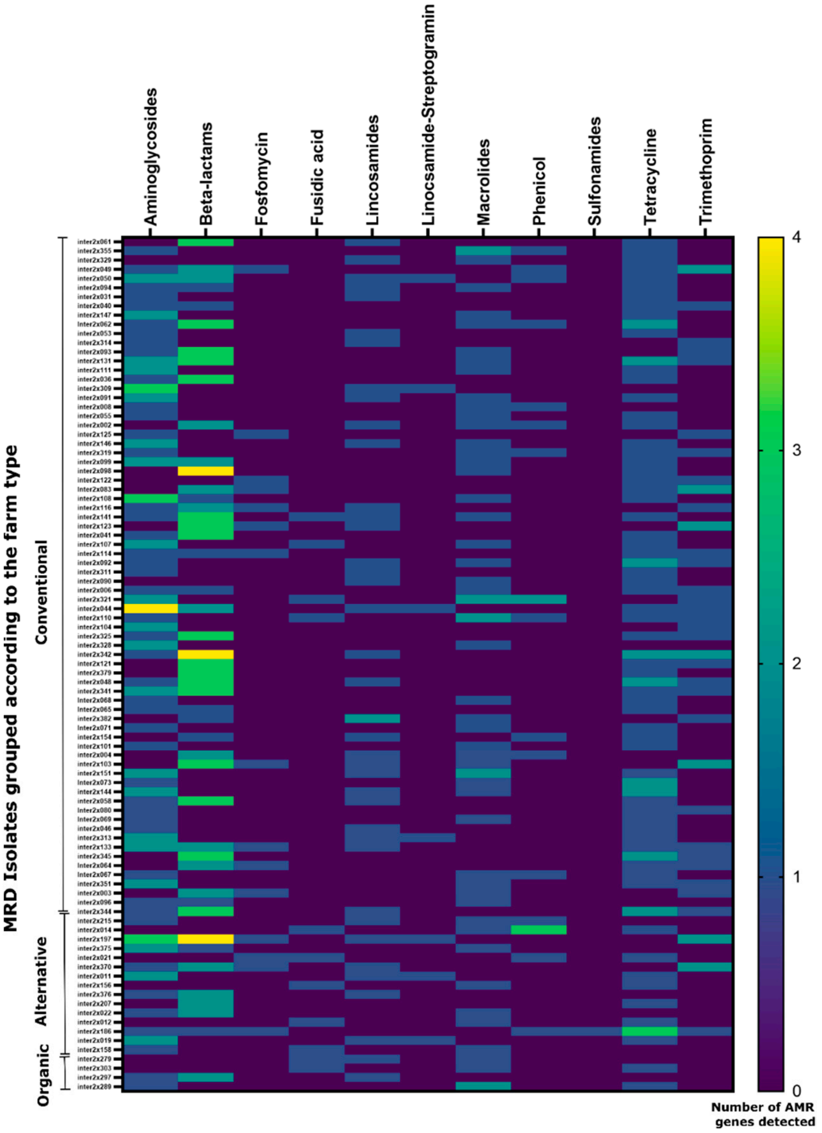

Figure S2. Heat map representation of the antimicrobial genes detected in MRD isolates. In the y-axis, the MRD isolates are classified according to farm type. The number of AMR genes detected in each antibiotic class is depicted by the legend in the right side.
