# Supplementary material for: Farming Practice Influences Antimicrobial Resistance Burden of Non-Aureus Staphylococci in Pig Husbandries
Source: Microorganisms. 2022 Dec 22;11(1):31. doi: 10.3390/microorganisms11010031 (PMC9865537; doi:10.3390/microorganisms11010031)
Supplement: Supplementary file 1 [file microorganisms-11-00031-s001.zip › Table S1_statistics_.pdf]

Table S1: Species distribution in the population ($p<0.03$) [*)]

NAS species	Number of isolates in each farm type			Number of isolates in each sample type		X^2	p	X^2	p	X^2	p
	Conventional (n=145)	Alternative (n=54)	Organic (n=41)	Pig commensal isolates (n=144)	Environmental isolates (n=96)	Conventional vs. alternative		Conventional vs. organic		Commensal vs. environmental	
S. arlettae	-	2	-	2	0	2.3409	0.126	NA	NA	0.18908	0.6637
S. chromogenes	6	6	-	1	11	2.2579	0.1329	0.67814	0.4102	11.875	0.0005689
S. cohnii	6	4	2	7	5	0.32935	0.566	2.1174e-30	1	3.6706e-30	1
S. epidermidis	11	3	-	13	1	0.03474	0.8521	2.083	0.1489	3.9803	0.04604
S. felis	1	-	-	1	0	1.3654e-31	1	8.5693e-32	1	5.8278e-31	1
S. haemolyticus	23	2	2	6	21	4.2461	0.03934	2.4376	0.1185	16.361	5.236e-05
S. hominis	5	-	-	0	5	0.76167	0.3828	0.43366	0.5102	5.3191	0.02109
M. lentus	-	3	2	5	0	4.8652	0.0274	3.2995	0.0693	1.9149	0.1664
S. muscae	1	-	-	1	0	1.3654e-31	1	8.5693e-32	1	5.8278e-31	1
S. pasteuri	12	1	1	7	7	1.7113	0.1908	0.89751	0.3435	0.25601	0.6129
S. pettenkoferi	1	-	-	1	0	1.3654e-31	1	8.5693e-32	1	5.8278e-31	1
S. piscifermentas	5	-	-	5	0	0.76167	0.3828	0.43366	0.5102	1.9149	0.1664
S. saprophyticus	-	1	1	0	2	0.26574	0.6062	0.45729	0.4989	1.0294	0.3103
M. sciuri	-	-	1	1	0	NA	NA	0.45729	0.4989	5.8278e-31	1
S. simulans	66	28	15	69	40	0.40482	0.5246	0.70572	0.4009	0.67302	0.412
S. warneri	7	1	-	7	1	0.29644	0.5861	0.93975	0.3323	1.5571	0.2121
S. xylosus	1	3	17	18	3	2.582	0.1081	0.93975	0.3323	5.2207	0.02232

[*)] Statistical analysis was performed using the 2-sample test for equality of proportions with continuity correction as described in Materials and Methods. Statistically significant results are highlighted in red. Borderline values are marked in green.
