# Supplementary material for: Farming Practice Influences Antimicrobial Resistance Burden of Non-Aureus Staphylococci in Pig Husbandries
Source: Microorganisms. 2022 Dec 22;11(1):31. doi: 10.3390/microorganisms11010031 (PMC9865537; doi:10.3390/microorganisms11010031)
Supplement: Supplementary file 1 [file microorganisms-11-00031-s001.zip › Table S3.pdf]

Supplementary Table S3

(i) Number of isolates displaying phenotypic and genotypic resistance for the antibiotic classes tested

Resistance details		Conventional farms (n=145)	Alternative farms (n=54)	Organic farms (n=41)	Resistance spectrum mediated by AMR gene
**Aminoglycosides**					
Phenotypic resistance	Gentamycin	2	0	0	
	Apramycin	0	0	0	
	Spectinomycin	22	7		
Genotypic resistance	*ant(9)-Ia*	6	4	0	Spectinomycin
	ant(6)-Ia	4	3	0	Streptomycin
	aaD1	16	1	0	Kanamycin, Tobramycin
	aac(6')-Ie/aph(2'')-Ia	1	0	0	Gentamycin, Amikacin, Tobramycin, Kanamycin
	spd	16	0	0	Spectinomycin
	spw	4	3	0	Spectinomycin
	at least one AMR gene	35	7	0	
**Beta-lactams**					
Phenotypic resistance		ND	ND	ND	
Genotypic resistance	*blaZ*	38	10	1	
	blaPC1	11	1	1	
	blaARL-1	0	2	0	
	at least one AMR gene	49	13	2	
**Cefoxitin/oxacillin resistance**					
Phenotypic resistance	Cefoxitin and Oxacillin (n=18)	15	2	1	
	Cefoxitin only (n=2)	1	1	0	
	Oxacillin only (n=13)	2	4	7	
Genotypic resistance	*mecA* (n=11)	10	1	0	
**Fosfomycin**					

Phenotypic resistance	Fosfomycin	57	9	9	
Genotypic resistance	*fosD*	0	1	3	
	fosB	11	3	0	
	at least one AMR gene	11	4	3	
**Fusidic acid**					
Phenotypic resistance	Fusidic acid	20	13	25	
Genotypic resistance	*fusD*	0	1	1	
	fusF	5	4	2	
	atleast one *AMR* gene	5	5	3	
**Macrolides (-lincosamides streptogramin B)**					
Phenotypic resistance	Erythromycin	56	11	4	
Genotypic resistance	*msrA*	5	4	3	Macrolides, Streptogramin B
	mphC	0	3	3	Macrolides
	ermT	1	0	0	Macrolides, Linosamides, Streptrogramin B
	ermC	45	1	1	
	ermA	5	4	0	
	erm43	0	0	1	
	atleast one AMR gene	52	12	5	
**Lincosamides (- streptogramins)**					
Phenotypic resistance	Clindamycin	81	17	6	
	Quinupristin	2	0	0	
Genotypic resistance	*vga(E)*	0	1	0	Linosamides, Streptrogramin A
	vga(L)C	7	0	0	
	vga(A)	12	2	4	
	sal(A)	0	0	1	
	lsa(E)	4	3	0	
	lnu(B)	4	3	0	Linosamides
	lnu(A)'	2	2	1	
	lnu(A)	10	1	4	
	at least one AMR gene	33	9	9	
**Phenicols**					
Phenotypic resistance	Chloramphenicol	15	6	0	

	Florfenicol	8	2	0	
Genotypic resistance	*cmx*	0	1	0	
	catA	7	4	0	
	fexA	8	2	0	
	at least one AMR gene	14	6	0	
**Sulfonamides**					
Phenotypic resistance		ND	ND	ND	
Genotypic resistance	*sul1*	0	1	0	
**Tetracycline**					
Phenotypic resistance	Tetracycline	77	12	13	
Genotypic resistance	*tet(33)*	0	1	0	
	tet(K)	64	11	3	
	tet(L)	18	0	0	
	tet(M)	11	0	1	
	tet(Z)	0	2	0	
	at least one AMR gene	80	11	4	
**Trimethoprim**					
Phenotypic resistance	Trimethoprim	11	1	2	
Genotypic resistance	*dfrC*	23	3	0	
	dfrD	5	0	0	
	dfrG	6	2	0	
	dfrK	6	0	2	
	at least one AMR gene	34	3	2	

(ii) Statistical analysis data of phenotypic and genotypic resistance distribution in the different farm types*).

	Conventional (n=145)	Alternative (n=54)	χ^2	p value	Organic (n=41)	χ^2	p value
**(a) Phenotypic resistance screening** (significance threshold p<0.0011)							
Cefoxitin	16	3	0.80682	0.3691	1	1.9027	0.1678
Oxacillin	17	6	6.2401e-31	1	8	1.0641	0.3023
Gentamicin	2	0	0.0046605	0.9456	0	3.1959e-31	1
Levofloxacin	2	0	0.0046605	0.9456	0	3.1959e-31	1
Erythromycin	56	11	5.0795	0.02421	4	10.901	0.000961
Clindamycin	81	17	8.4074	0.003737	6	20.197	6.985e-06
Linezolid	1	0	1.3654e-31	1	1	0.010287	0.9192
Daptomycin	0	0	NA	NA	1	0.45729	0.4989
Teicoplanin	6	0	1.1062	0.2929	0	0.67814	0.4102
Vancomycin	0	0	NA	NA	0	NA	NA
Tetracycline	77	12	13.955	0.0001873	13	5.0336	0.02486
Tigecycline	1	0	1.3654e-31	1	0	8.5693e-32	1
Fosfomycin	57	9	8.1086	0.004406	9	3.4831	0.062
Fusidic acid	20	13	2.3092	0.1286	25	36.267	1.721e-09
Rifampicin	0	1	0.26574	0.6062	0	NA	NA
Trimethoprim	11	1	1.3834	0.2395	2	0.064326	0.7998
Apramycin	0	0	NA	NA	0	NA	NA
Chloramphenicol	15	6	7.1608e-31	1	0	3.3236	0.06829
Florfenicol	8	2	0.024289	0.8762	0	1.2134	0.2707
Spectinomycin	22	7	0.02785	0.8675	0	5.6753	0.01721
Quinupristin	2	0	0.0046605	0.9456	0	3.1959e-31	1
Ciprofloxacin	6	0	1.1062	0.2929	0	0.67814	0.4102
**(b) Genotypic resistance screening** (significance threshold p<0.0022)							
Aminoglycoside	35	7	2.318	0.1279	0	10.662	0.001094
Beta-lactams	49	13	1.3093	0.2525	2	12.014	0.0005279
Cefoxitin/oxacillin resistance	10	1	1.0731	0.3002	0	1.7864	0.1814

Fosfomycin	11	4	3.5561e-31	1	3	3.7204e-31	1
Fusidic acid	5	5	1.6995	0.1924	3	0.41237	0.5208
Macrolides (-lincosamides - streptogramin B)	52	12	2.7592	0.0967	5	7.3466	0.006719
Lincosamides (- streptogramins)	33	9	0.54926	0.4586	9	5.7516e-31	1
Phenicols	14	6	0.0014926	0.9692	0	3.006	0.08295
Sulfonamides	0	1	0.26574	0.6062	0	NA	NA
Tetracycline	80	11	17.826	2.421e-05	4	24.818	6.301e-07
Trimethoprim	34	3	7.1823	0.007363	2	5.922	0.01495
**(c) Multiple resistance determinant (MRD) analysis** (significance threshold $p<0.05$)							
Phenotypic MRD isolates	74	18	4.2731	0.03872	10	8.1178	0.004383
Genotypic MRD isolates	74	15	7.6935	0.005542	4	20.703	5.363e-06

[*)] Statistical analysis was performed using the 2-sample test for equality of proportions with continuity correction as described in Materials and Methods. Alternative and organic farms were compared each with conventional farms as the baseline. Statistically significant results are highlighted in red. Borderline values are marked in green.
